# The protective role of Kombucha extract on the normal intestinal microflora, high-cholesterol diet caused hypercholesterolemia, and histological structures changes in New Zealand white rabbits

**Published:** 2020

**Authors:** Zeytoon Alaei, Monir Doudi, Mahbubeh Setorki

**Affiliations:** 1 *Department of Microbiology, Falavarjan Branch, Islamic Azad University, Isfahan, Iran*; 2 *Department of Microbiology, Falavarjan Branch, Islamic Azad University, Isfahan, Iran*; 3 *Department of Biology, Izeh Branch, Islamic Azad University, Izeh, Iran*

**Keywords:** Kombucha extract, Intestinal microflora, Hypercholesterolemia, Arteriosclerosis, FBS, New Zealand white rabbits

## Abstract

**Objective:**

The aim of the current study was to investigate the effect of Kombucha extract (tea) on the normal intestinal microflora and histological structures in rabbits.

**Materials and Methods:**

This study was a descriptive-analytical investigation. Thirty-two male New Zealand rabbits were randomly divided into 4 groups as follows: Normal diet (I), high-cholesterol diet (II), normal diet plus Kombucha extract (II), and high-cholesterol diet plus Kombucha extract (IV). Microbial cultures were taken from feces of rabbits before and after the applied treatments. The rabbits' blood was collected from the heart to determine the level of cholesterol, glucose and iron in the blood. Aorta and coronary heart microtome cut samples were prepared for detection of histological changes.

**Results:**

Rabbit stool cultures before treatment with Kombucha extract included *Enterobacter aerogenes*,* Providencia rettgeri*, *Proteus mirabilis*, *Pseudomonas aeruginosa* and *Klebsiella oxytoca*. However, *Escherichia coli*, *Enterobacter aerogenes*, *Pseudomonas aeruginosa*, *klebsiella pneumoniae* and *Hafnia alvei *were found in stool cultures after treatment with Kombucha extract. Group IV had significantly lower blood cholesterol levels. Animals that received Kombucha extract only had lower fasting blood sugar (FBS) levels. Healthy rabbits that received Kombucha extract only and group (IV) showed a significant increase in iron (Fe) levels and a significant decrease in total iron binding capacity (TIBC) levels. In both groups III and IV, the right and left coronary arteries were completely normal and no lesions were observed in the intima.

**Conclusion:**

The results of this study showed minor changes in the intestinal microflora of rabbits after treatment with Kombucha extract and positive effects of this tea on some risk factors (hypercholesterolemia, arteriosclerosis, and FBS).

## Introduction

Cardiovascular disease is one of the leading causes of death in the world and is expected to kill 25 million people by 2020 (Gang et al., 2018 ▶). Atherosclerosis is a major risk factor for the development of cardiovascular disease. Oxidation of lipoproteins and elevated serum cholesterol levels play an important role in the pathogenesis of atherosclerosis, so it has been suggested that inhibition of low-density lipoprotein (LDL) oxidation and reduced triglyceride, cholesterol and LDL levels delay atherosclerosis (Ceriello et al., 1999). So, compounds with antioxidant activity and hypocholesterolemic effects are expected to be useful in this context (Brownlee, 2001 ▶). Kombucha is an ancient nutritional and healing source of Asian origin. Due to the growing attention of Western societies to Eastern therapeutics, Kombucha reached Eastern Europe via Russia. In these areas, Kombucha was considered a cheerful and healing drink that balanced the diet. This potion exhibited miracles along its way to the Western world. Due to its beneficial effects against new diseases, it was given names such as magic mushroom, miracle mushroom and the elixir of youth and longevity (Burtis and Ashwood, 1999 ▶).

Kombucha is a beverage made from fermented black tea and contains ethanol, carbon dioxide, high concentrations of organic acids (gluconic and glucuronic, acetic and lactic acids) as well as a number of other metabolites including vitamins B1, B2, B3, B6, B12, and C and usnic acid. Usnic acid found in Kombucha tea has strong antibacterial and antiviral properties. Gluconic acid is known as the main compound of Kombucha tea and is capable of eliminating the effects of all toxins produced by pathogenic microbes in the body (Marsh et al., 2014 ▶). In fact, Kombucha is a traditional refreshing beverage that is prepared by the fermentation of sweet tea using powerful coexistence of acetic acid bacteria such as *Acetobacter xylinum*, *Acetobacter aceti*, and *Bacterium gluconicum *with yeasts such as *Saccharomyces kudriavzevii*, *schizosaccharomyces pombe*, and *Saccharomyces cerevisiae*, and *Torulopsis*, *Brettanomyces *and* Pichia *spp. (Battikh et al., 2012 ▶; Greenwalt et al., 2000 ▶). This strong collaboration between several species of bacteria and yeast leads to inhibition of the growth of pathogenic and contaminating bacteria. However, the biological growth pattern of these microorganisms during fermentation has not yet been studied. Yeasts convert sugar to ethanol and ethanol is oxidized to acetic acid by *Acetobacter* strains, thereby lowering the pH of the solution. Recent research has shown that the antimicrobial activity of Kombucha against pathogenic microorganisms is largely related to acetic acid content. The attractive end product is an acidic, sour and slightly carbonated beverage with sugars, organic acids, tea compounds, vitamins and minerals (Velićanski et al., 2013 ▶). The results of a new study published in the journal Nature showed the role of gut bacteria in maintaining arterial health (Borody et al., 2012 ▶). Numerous studies have suggested that gut bacteria play an important role in healthy aging (Borody et al., 2012 ▶). The purpose of this study was to evaluate the effect of Kombucha extract on the normal intestinal microflora and a number of blood cholesterol-induced biochemical factors changes in male New Zealand white rabbits and investigate the cardiac histological structures in these rabbits.

## Materials and Methods

**Kombucha extract (tea) preparation**

An amount of 60 g of natural sugar (Kamvar, Iran) was added to about 800 ml purified municipal boiled water and boiled for 2 to 3 min. Then, 30 g of standard Gilan high-quality dry tea was added and the mixture was filtered shortly after cooling and transferred to a sterilized glass container. Then, 100 g of washed and sterilized mushrooms in addition to 0.9% normal saline was added to the container. The container was then covered with a clean, sterilized linen cloth to prevent contaminants entering and transferred to an incubator at 27°C for one week. Finally, the fermentation product was separated under aseptic conditions and transferred to a 4°C refrigerator. The product was used as Kombucha extract (Murugesan et al., 2009 ▶).

**Animals and grouping**

In the present experimental study, 32 New Zealand rabbits (weighing 2000- 2500 g) were obtained from Pasteur Institute of Tehran. The rabbits were kept at the temperature of 21±2°C in a period of 12 hr light and 12 hr dark with free access to water and food. Animals were divided into 4 groups of 8 animals each. Group I received standard diet for 40 days. Group II (hypercholesterolemia) received high-cholesterol diet (1%) for 40 days. Group III received standard diet with Kombucha extract 10 ml/kg daily by oral gavage method for 40 days. Group IV received an oral daily high-cholesterol diet with Kombucha extract for 40 days. At the end of the experiment, the animals were deeply anesthetized, blood samples were collected and biochemical blood tests to measure cholesterol, Fe and FBS levels, were done.

**Culturing and biochemical identification of rabbit’s intestinal microflora**

First, the rabbit’s stools suspensions before and after treatment with Kombucha extract were prepared in normal saline. Then, the supernatants were separated by centrifugation and kept overnight at 37°C. Finally, the supernatants were cultured on Nutrient agar (Himedia, India; NA) and MacConkey agar (Himedia, India; MC) media and incubated overnight at 37°C. The isolates were streaked on NA and MC media and incubated overnight at 37°C for purification. Then, all colonies were evaluated for their macroscopic appearance, such as colony color, margin, shape, size and state. Then, the colonies were stained by Gram-staining for microscopic analysis of the shape, arrangement and Gram reaction of the bacterial cells (Fann et al., 2001 ▶).

**Histological analysis**

At the end of experiment, the animals were killed, the aorta and coronary artery were removed and tissue sections (5 μm) aorta fixed by immersion at room temperature in 10% formalin solution stained for fat deposits. Secondly, for tissue dehydration, a series of low-to-high concentrations of alcohol solutions was used. Thirdly, alcohol removing and tissue clearing were done by using xylenol or acetone. In the fourth stage, the tissues were put into aluminum or steel molds and molten paraffin was poured on them. Paraffin molds were placed in the refrigerator for 24 hr. Finally, after solidification, tissues were cross-sectioned using a microtome to sections with 10 µm thickness and stained by hematoxylin and eosin staining protocol (Chekanov, 2003 ▶).

**Atherosclerotic plaque grade evaluation**

After staining of coronary and aorta with hematoxylin and eosin, atherosclerotic plaque grade (graded 1-4) was determined. Grade 1 included plaque thickness less than half of media thickness, mild forms of endothelial dysfunction, increased plasma permeability for lipids, some degrees of blood cells (macrophage and platelet) adhesions to endothelial cells, and the presence of macrophage and foamy cell in the intima. Grade 2 included plaque thicknesses half of the media thickness, and macrophage and smooth muscle cells presence in the plaque. Grade 3 included plaque thickness as much as media thickness, the presence of macrophages and smooth muscle cells within the plaque, indicating the synthesis and proliferation of the extracellular matrix by the smooth muscle and abundant connective tissue within the plaque accumulating collagen and proteoglycan. In grade 4, plaque thicknesses were more than media thickness, and the plaque was formed as a large, fully protruding lipid nucleus at the endothelial surface. Also, filtration of inflammatory cells including macrophages and palpable in addition to calcification in the lipid nucleus, were seen (Chekanov, 2003 ▶).

**Statistical analysis**

To compare the serum biochemical findings, the differences between the data before and after was determined and considered independent variable of variations. One-way ANOVA (LSD test) was used to analyze the differences among the experimental groups. A value of p<0.05 was considered statistically significant. The data is expressed as mean±S.E.M.

## Results

**Rabbit intestinal bacteria (stool culture) before treatment with Kombucha extract**

The results (Tables 1 and 2 showed the presence of *Enterobacter aerogenes*, *Providencia rettgeri*, *Proteus mirabilis*, *Pseudomonas aeruginosa* and *Klebsiella oxytoca *in stool culture before treatment with Kombucha extract. The macroscopic and microscopic properties of the above mentioned bacteria are shown in Table 1 and the results of biochemical tests on the bacteria are presented in Table 2.

**Table 1:** Macroscopic and microscopic characteristics of intestinal bacterial microflora (stool culture) of rabbits before treatment with Kombucha extract

**Bacterial sp.**	**Macroscopic characteristics **	**Microscopic characteristics**
*Enterobacter aerogenes*	Mucoid, small, Circular, Cream colored colonies	Encapsulated short Gram-negative rods
*Providencia rettgeri*	Mucoid, small, Circular, Cream colored colonies	Flagellated Gram-negative rods
*Proteus mirabilis*	Rough, Medium sized, Circular, Cream-orange colored colonies	Flagellated short Gram-negative coccobacilli
*Pseudomonas aeruginosa*	Smooth, Medium sized, Circular, Cream colored colonies; Green-blue colonies on Mueller Hinton Agar (MHA) and Cream colored colonies on MacConkey agar (MCA)	Largesized Gram-negative streptobacilli
*Klebsiella oxytoca*	Mucoid, small, Cream colored colonies; Pink colored colonies on MCA and red-purple colonies on Eosin Methylene Blue Agar (EMB)	Medium sized Gram-negative coccobacilli

**Table 2:** The results from biochemical tests on intestinal bacterial microflora (stool culture) of rabbits before treatment with Kombucha extract

***Klebsiella oxytoca***	***Pseudomonas aeruginosa***	***Providencia rettgery***	***Proteus mirabilis***	***Enterobacter aerogenes***	**Bacterial sp./ Biochemical tests**
-	*	-	-	-	Arginine dihydrolase
+	*	-	-	+	Lysine decarboxylase
-	*	-	+	+	Ornithine decarboxylase
+	+	+	-	+	Manitol fermentation
+	-	-	+	+	Sucrose fermentation
+	-	-	-	+	Lactose fermentation
+	-	+	+	+	Glucose fermentation
-	+	+	+	+	Motility
+	-	+	-	-	Endol production
-	-	-	+	-	SH2 production
A/A	K/K	K/A	K/A; gas	K/A; gas	Growth on Triple sugar Iron agar
+	+	+	+	+	Citrate utilization
+	-	-	+	+	Voges–Proskauer test
+	-	+	+	-	Methyl red test
+	+/-	+	+	+	Urease
-	+	-	-	-	Oxidase
+	+	+	+	+	Catalase
*	Oxidative	*	*	*	Oxidative/Fermentative test
*	Green-blue	-	-	-	Pigment

(+) positive result, (-) negative result, (*) not ordinary for identification of the bacterium

**Rabbit intestinal bacteria (stool culture) after treatment with Kombucha extract**

The results of biochemical tests on the bacteria are presented in Table 3. *Escherichia coli*, *Enterobacter aerogenes*, *Pseudomonas aeruginosa*, *Klebsiella pneumoniae* and *Hafnia alvei *were identified in rabbit feces cultures after treatment with Kombucha extract.

**Table 3:** The results from biochemical tests on intestinal bacterial microflora (stool culture) of rabbits after treatment with Kombucha extract

***Hafnia alvei***	***klebsiella pneumoniae***	***Pseudomonas aeruginosa***	***Enterobacter aerogenes***	***Escherichia coli***	**Bacterial sp./ Biochemical tests**
-	-	*	-	-	Arginine dihydrolase
+	+	*	+	+	Lysine decarboxylase
+	-	*	+	+	Ornithine decarboxylase
+	+	+	+	+	Manitol fermentation
+	+	-	+	-	Sucrose fermentation
-	+	-	+	+	Lactose fermentation
+	+	-	+	+	Glucose fermentation
+	+	+	+	+	Motility
-	-	-	-	+	Endol production
-	-	-	-	-	SH2 production
A/A	A/A; gas	K/K	K/A; gas	A/A; gas	Growth on Triple sugar Iron agar
-	+	+	+	-	Citrate utilization
+	+	-	+	-	Voges–Proskauer test
+	+	-	-	+	Methyl red test
-	+	+/-	+	+/-	Urease
-	-	+	-	-	Oxidase
+	+	+	+	+	Catalase
*	*	Oxidative	*	*	Oxidative/Fermentative test
*	-	Green-blue	-	-	Pigment

(+) positive result, (-) negative result, (*) not ordinary for identification of the bacterium.

**Biochemical factors results**

**Figure 1:**
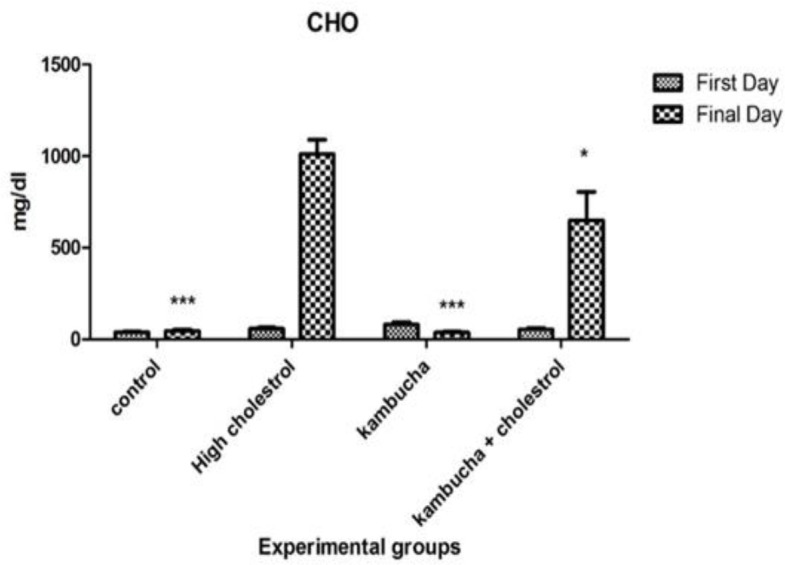
Effect of Kombucha extract on serum cholesterol levels in rabbits that received standard and high-cholesterol diet. *indicates a significant difference between the control group and the high-cholesterol diet group (p<0.05), ***indicates a significant difference between the control group and the high-cholesterol diet group (p<0.001).

According to the results presented in Figure 1, cholesterol level in rabbits that received high-cholesterol diet was significantly higher than the control group after 40 days of treatment with standard diet. In addition, in rabbits that received high-cholesterol diet with Kombucha extract, the blood cholesterol level was significantly lower than the high-cholesterol diet group.

**Figure 2:**
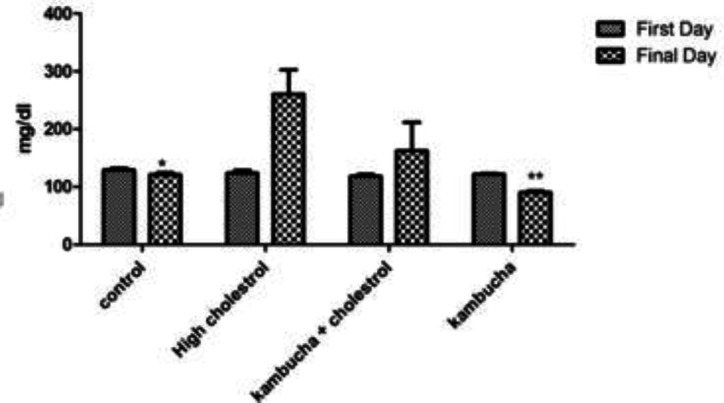
Effect of Kombucha extract on serum FBS levels in rabbits that received standard or high-cholesterol diet. *indicates a significant difference between the control group and the high-cholesterol diet group (p<0.05), **indicates a significant difference between the control group and the high-cholesterol diet group (p<0.01).

The results shown in Figure 2 indicate that rabbits that received high-cholesterol diet had significantly higher blood FBS levels than the control group. Animals that received Kombucha extract only had significantly lower FBS levels compared to the hypercholesterolemia group.

**Figure 3:**
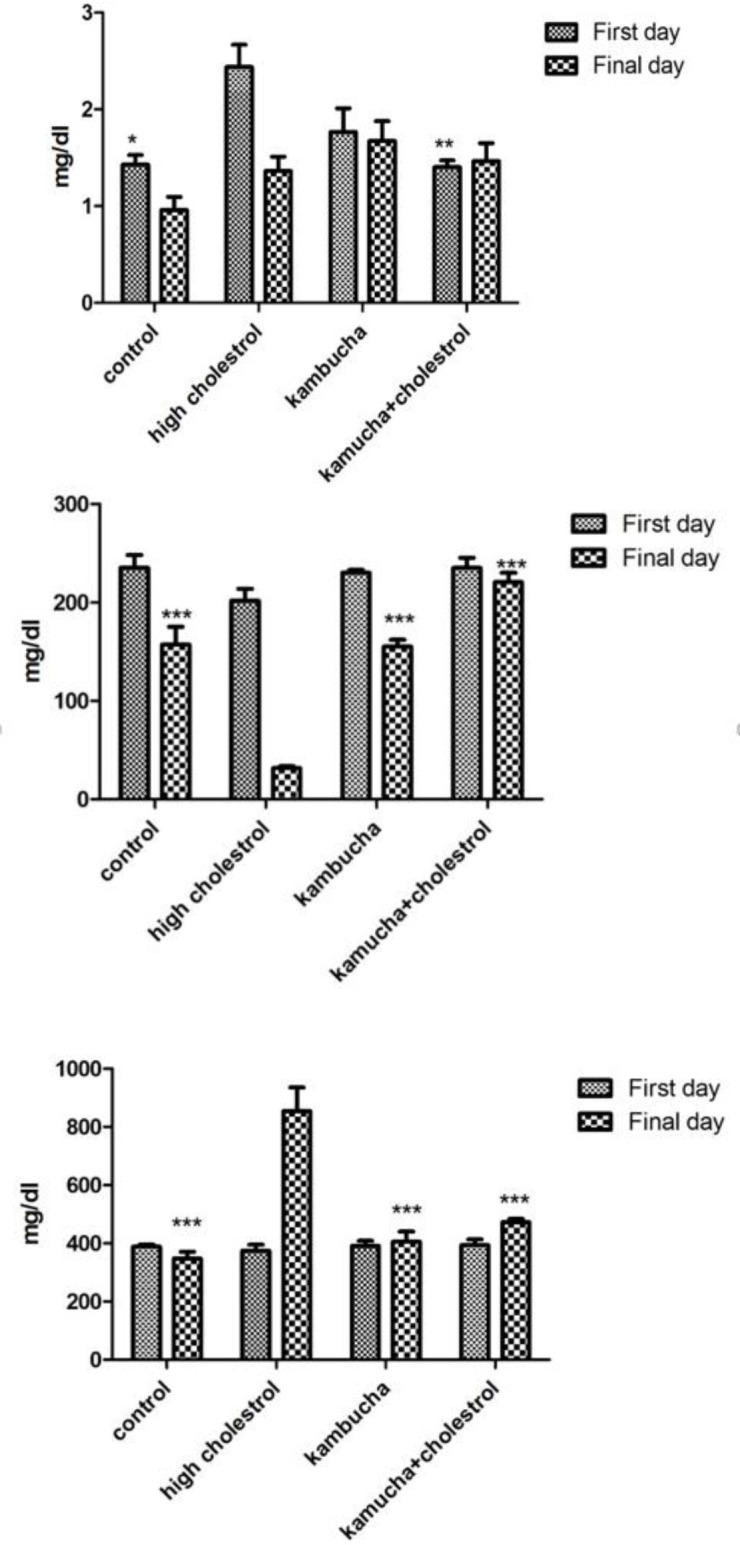
Effect of Kombucha extract on serum Fe, ferritin, and TIBC levels in rabbits that received standard or high-cholesterol diet. ***indicates a significant difference between the control group and the high-cholesterol diet group (p<0.001). *indicates a significant difference between the control group and the high-cholesterol diet group (p<0.05), **indicates a significant difference between the control group and the high-cholesterol diet group (p<0.01).

The results of blood Fe, ferritin, and total iron binding capacity (TIBC) levels are shown in Figures 3A, 3B and 3C, respectively. According to these results, rabbits that received high-cholesterol diet for 40 days, showed a significant decrease in blood Fe level compared with the control group.

Healthy rabbits that received Kombucha extract only and rabbits that received high-cholesterol diet with Kombucha extract showed significant increases in blood Fe levels when compared with the hypercholesterolemia groups. The results of ferritin level showed that on the first day of the experiment, the hypercholesterolemia group had a statistically higher level of ferritin compared to the control group. The hypercholesterolemia group that received Kombucha extract also had a significant decrease in blood ferritin level on the first day of testing compared with the hypercholesterolemia group. TIBC was significantly higher in the hypercholesterolemia group 40 days after the initiation of the experiment compared to the control group. In the groups that received Kombucha extract alone and Kombucha extract with cholesterol, TIBC decreased significantly 40 days after treatment compared to the hypercholesterolemia group.

**Histological test results**

**Figure 4:**
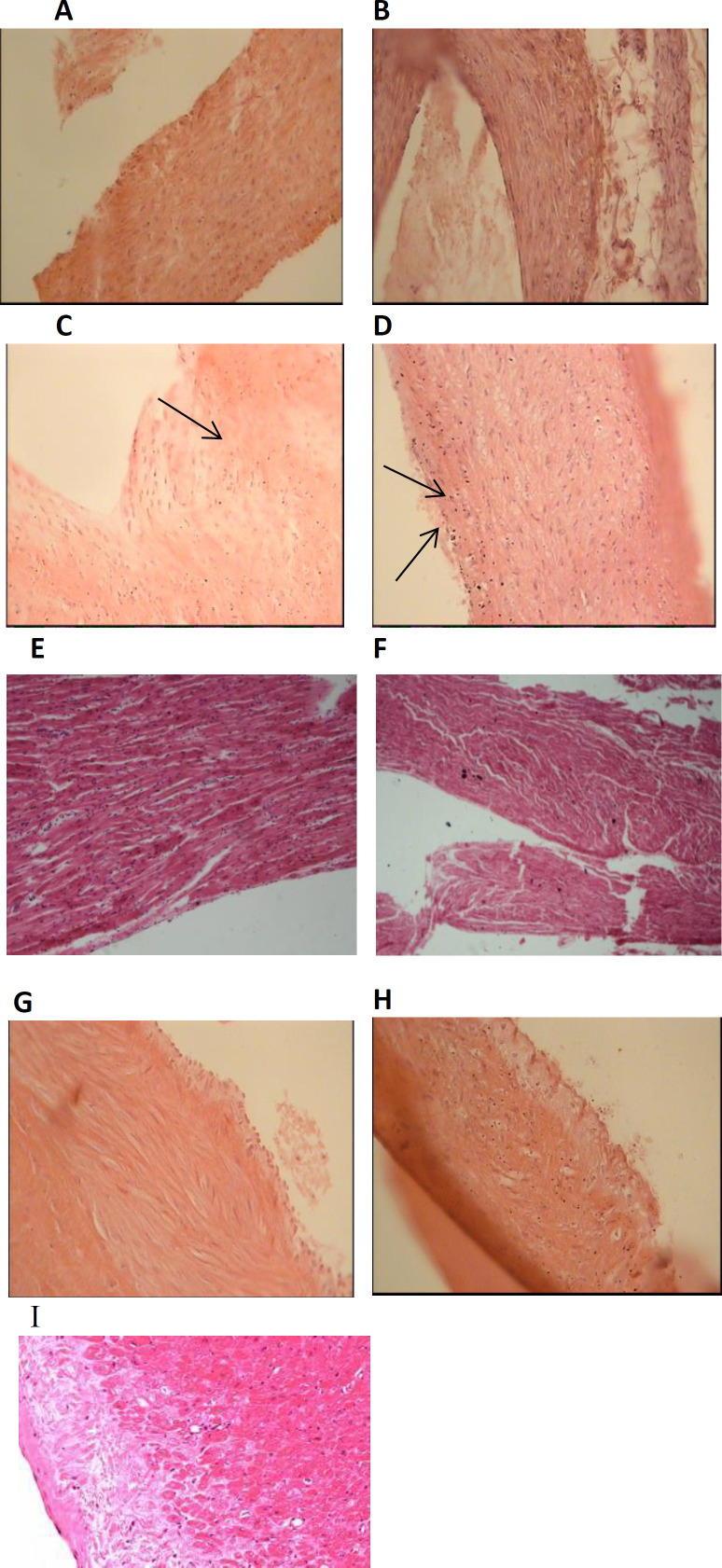
Histopathological changes of right and left coronary arteries in New Zealand rabbits that received standard diet (A, B), high-cholesterol diet (C, D), normal diet with Kombucha extract (E, F), high-cholesterol diet with Kombucha extract (G,H ) for 40 days, I: standard coronary artery(H&E staining); x40 magnification)

Histological analysis of the changes as the result of cholesterol consumption in arterial coronary walls (right and left) sections were compared in experimental groups. The results showed the normal right (Figure 4A) and left (Figure 4B) coronary vessels with no lesions in the intima in the normal diet group.

Atherosclerotic plaques with the thickness as media and the degree of 3, were detected in sections prepared from right (Figure 4C) and left (Figure 4D) coronary arteries in the hypercholesterolemia (1%) group. In these plaques, the macrophages that were full of lipids produced foamy cells. In both normal diet and high-cholesterol diet groups that received Kombucha extract, the right (Figures 4E and 4G) and left (Figure 4F and 4H) coronary arteries were quite normal and no lesions were seen in the intima.

**Aortic histological analysis**

The aortic sections were examined histologically and the changes in the aorta wall caused by cholesterol consumption, were compared among the experimental groups. As it is clear in Figure 2A, in the control group (normal regimen), the vessel is completely normal and no lesion is seen in intima and media. Atherosclerotic plaques were detectable in aortic sections of cholesterol (1%) treated group. Plaque thickness was more than half the thickness of the media and the plaque degree was 3. In this plaque, macrophages that were full of lipids produced foamy cells (Figure 2B). The aortic vessel in the normal diet and high-cholesterol diet groups that received Kombucha extract, was quite normal and no lesions were seen in the intima.

**Figure 5:**
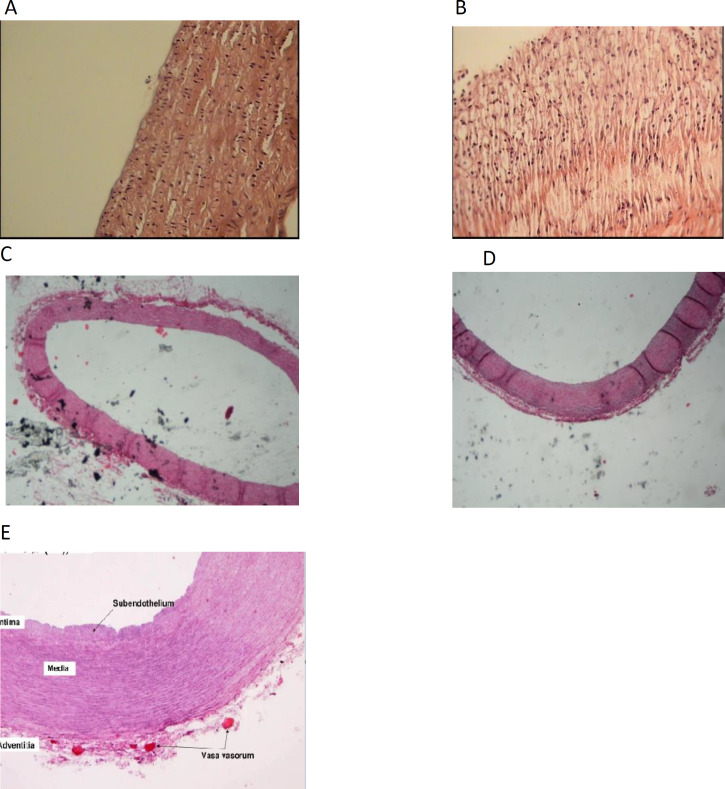
Histopathological changes of Aorta artery in New Zealand rabbits that received standard diet(H&E staining); x40 magnification) (A), high-cholesterol diet(H&E staining); x40 magnification) (B), normal diet with Kombucha extract (C), high-cholesterol diet with Kombucha extract(H&E staining); x4 magnification ) (D ) for 40 days(H&E staining); x4 magnification), E: standard aorta(H&E staining); x40 magnification).

## Discussion

Coronary heart disease is often caused by coronary artery obstruction due to arteriosclerosis. The association between plasma lipid abnormalities and the risk of coronary artery disease is well known. In addition to quantitative disorders such as elevated cholesterol, plasma lipid quality disorders plays an important role in the pathogenesis of atherosclerosis. Elevated cholesterol levels and in some cases triglycerides as risk factors for the development of atherosclerosis, are also risk factors for cardiovascular diseases (Hirunpanich et al., 2006 ▶). The results of this study showed that consumption of high-cholesterol diet (1%) for 40 days in male New Zealand White rabbits significantly increased cholesterol and FBS levels in the high-cholesterol group compared to the normal diet group. Also, Kombucha extract treatment significantly reduced blood cholesterol and FBS levels. Evidence suggests that oxidation of low-density lipoproteins (LDLs) plays an important role in atherogenesis, leading to the belief that catalytic iron may also be involved in enhancement of the formation of free radical species in the atherogenesis process. Also, elevated blood iron levels were observed in human and animal atherosclerotic lesions (Pinheiro et al., 1996 ▶). In two experimental studies, elevated blood iron increased oxidative stress in rats and increased atherosclerotic lesions in hypercholesterolemic rabbits (Dabbagh et al., 1994 ▶; Araujo et al., 1995 ▶), but in another similar study, a decrease in blood iron level was observed in a rabbit model of atherosclerosis (Dabbagh et al., 1997 ▶). Also, elevated blood plasma iron could not lead to increased LDL oxidation *in vitro* (Bergeret al., 1997 ▶). The results of our study were in agreement with recent studies and showed that hypercholesterolemic rabbits had significant decreases in blood iron and treatment with Kombucha extract for 40 days, led to significant recompense of this reduction (Ola G, 2014 ▶; Araujo et al., 1995 ▶).

The oxidative stress burden plays an important role in the development of the disease and complications of increased cholesterol or hypercholesterolemia. Therefore, it is likely that antioxidants may be associated with the elimination of oxidative effects that lead to high cholesterol disease. Studies showed high levels of polyphenols in Kombucha tea. It was shown that the amount of polyphenols increased during the fermentation process, which could be due to the activity of bacterial and yeast enzymes during fermentation that convert complex polyphenolic compounds into small molecules with antioxidant potential (Jayabalan et al., 2007 ▶).

Fermentation fluid analysis showed that acetic acid, lactic acid and gluconic acid are the main chemical constituents of Kombucha tea and it has been reported that gluconic acid is the main therapeutic agent in Kombucha tea that acts as a detoxifying agent in the liver. Gluconic acid seems to be one of the key ingredients of Kombucha tea. Also, polyphenols and organic acids were active components in Kombucha tea that showed beneficial effects and health benefits (Lončar et al., 2000 ▶).

Histological results showed that in both normal diet and hypercholesterolemic diet groups that received Kombucha extract, no lesions were seen in the coronary artery intima, and in these groups, Kombucha extract led to normal aortic vein so it was completely normal and no lesions were seen in the intima. Positive histopathological effects of Kombucha tea on pancreatic liver tissue have also been observed in other studies (Aloulou et al., 2012 ▶).

Researchers have shown that the microflora of the rabbit gastrointestinal tract vary with age, diet, and use of antibiotics and interfering substances. It was suggested that the gastrointestinal microflora of the young rabbit can often contain a variety of *Streptococcus *species and *Enterobacteriaceae* (Fann et al., 2001 ▶). In the present study, the intestinal microflora of male New Zealand rabbits purchased from the Pasteur Institute of Tehran, Iran, in the early days before treatment with Kombucha black extract was most related to the family of Enterobacteriaceae (*Enterobacter aerogenes*,* Providencia rettgeri*,* Proteus mirabilis*, and *Klebsiella oxytoca*) and one spp. of *Pseudomonadaceae* family (*Pseudomonas aeruginosa*). No Gram-positive bacteria were isolated from the intestine of these rabbits in stool culture. The differences between the results of this study and other researches in rabbit's gut natural microflora can be due to different reasons such as the rabbit types, and age, and diet and geographical factors such as a country's climate and many other factors. But the results showed that the microflora of rabbits' intestines after consuming Kombucha extract shifted to other beneficial bacteria, such as *Escherichia coli *which is a potent lactose fermenting bacterium. The presence of this bacterium in the stomach and intestine of rabbits has advantages such as production of vitamins B, especially B6 in the stomach and pathogenesis disadvantages caused by production of aggressins, capsule, and toxins (Fann et al., 2001 ▶). However, by comparing the initial natural microflora of the rabbits' intestines before treatment with Kombucha extract and the same microflora after the treatment, it was revealed that the flora changed to some extent, but without any major alterations. In the former, more than 95% of the bacteria isolated from rabbit stool cultures belonged to the *Enterobacteriaceae* family whereas post-treatment, although the pattern of the genera and species of bacteria changed somewhat, again, 95% of the bacterial genera belonged to the *Enterobacteriaceae* family, although the persistent presence of *Pseudomonas* *aeruginosa* in both pre and post treated rabbits extractcan propose this bacterium as a constant microflora of the rabbit intestine. The microflora of rabbits’ intestines was also slightly different before and after treatment with Kombucha extract. However, both constant and transient bacterial microflora of rabbits (fecal culture) was Gram-negative bacteria. 

The results of this study showed minor differences in the intestinal microflora of male New Zealand rabbits before and after treatment with Kombucha extract. The findings of the present study also showed that Kombucha extract had the potential to prevent cholesterol elevation probably due to the presence of substances such as polyphenols, gluconic, glucuronic, and lactic acids and a variety of vitamins, amino acids, antibiotics and some micronutrients produced during fermentation. Also because the antioxidant activity that was proposed for this natural product, it can be used as a therapeutic or pragmatic nutrient.
